# Proton beam therapy as an effective treatment option for recurrent endometrial cancer

**DOI:** 10.1016/j.gore.2025.102009

**Published:** 2025-12-18

**Authors:** Yuta Endo, Yoshiaki Takagawa, Yuki Yoshimoto, Koki Ando, Rei Nishikawa, Masanori Machida, Yuntao Dai, Ichiro Seto, Motohisa Suzuki, Takahiro Kato, Shigenori Furukawa, Shu Soeda, Keiya Fujimori, Masao Murakami

**Affiliations:** a Department of Gynecology, Southern TOHOKU General Hospital, Fukushima, Japan; b Department of Regional Gynecologic Oncology, Fukushima Medical University School of Medicine, Fukushima, Japan; c Southern TOHOKU Proton Therapy Center, Fukushima, Japan; d Department of Radiation Oncology, Southern TOHOKU General Hospital, Fukushima, Japan; e Department of Minimally Invasive Surgical and Medical Oncology, Fukushima Medical University School of Medicine, Fukushima, Japan; f Department of Radiological Sciences, School of Health Sciences, Fukushima Medical University, Fukushima, Japan; g Department of Obstetrics and Gynecology, Fukushima Medical University School of Medicine, Fukushima, Japan

**Keywords:** Recurrent endometrial cancer, Proton beam therapy, Radiation therapy

## Abstract

**Objective:**

To evaluate the efficacy and safety of proton beam therapy (PBT) for recurrent endometrial cancer (REC) and to identify predictors of local control (LC).

**Methods:**

We retrospectively reviewed REC patients treated with PBT at our institution between 2008 and 2021. LC, progression-free survival (PFS), and overall survival (OS) were estimated using the Kaplan–Meier method, and prognostic factors were analyzed. Toxicities were graded according to CTCAE v5.0.

**Results:**

Nine patients with a total of 14 lesions were included (median follow-up, 36.4 months). The 1- and 2-year LC rates were 80.2 % and 68.8 %, respectively. Median PFS and OS were 13.6 and 36.4 months, respectively. Tumor size significantly influenced LC, with lesions < 22.5 mm showing superior outcomes. No grade ≥ 3 toxicities attributable to PBT were observed.

**Conclusions:**

These findings suggest that PBT may be a safe and effective salvage treatment for REC, particularly in patients with smaller tumors.

## Highlights

• Proton beam therapy achieved favorable local control with minimal toxicity in recurrent endometrial cancer.
• Tumor size was the only significant predictor of local control, with smaller tumors showing better outcomes.
• PBT may be a suitable salvage option, particularly for patients with limited-volume recurrence.

## 1 Background

Endometrial cancer is the most common gynecologic malignancy with increasing incidence and mortality. The primary treatment is surgery including total hysterectomy with bilateral salpingo-oophorectomy and/or retroperitoneal lymph nodes dissection, sometimes accompanied by adjuvant therapy with radiation therapy (RT), chemotherapy, or a combination of both (de Boer et al., 2018, Nomura et al., 2019). However, approximately 4–20 % of patients develop locoregional recurrence, with higher rates among those with advanced disease, and treatment options depend on previous therapies and recurrence location (Rütten et al., 2021).

RT plays a crucial role in managing recurrent endometrial cancer (REC), particularly for patients who have not received prior RT. Previous studies demonstrated the efficacy of salvage RT for REC (Arden et al., 2020; Bradford et al., 2015). Proton beam therapy (PBT) is an advanced form of RT that enables precise dose delivery while minimizing exposure to surrounding normal tissues (Levin et al., 2005). Compared with photon-based techniques such as intensity-modulated RT (IMRT) and stereotactic body RT, PBT provides a distinct dosimetric advantage due to the Bragg peak, allowing for rapid dose fall-off beyond the target volume. From a logistical standpoint, SBRT is typically delivered in a small number of high-dose fractions, whereas IMRT and PBT are commonly administered over more conventional fractionation schedules, with PBT offering the potential for dose escalation or re-irradiation while maintaining organ-at-risk constraints (Jacobson and Galvan-Turner, 2020, Levin et al., 2005). This difference is particularly relevant in REC, where lesions often arise near radiosensitive structures. In addition, a prospective comparative study has shown that adjuvant PBT is associated with reduced gastrointestinal (GI) toxicity compared with IMRT for uterine cancer, while maintaining oncologic outcomes (Anderson et al., 2023). However, the clinical efficacy of PBT for REC remains unclear. Several case reports and a case series have described favorable outcomes, showing complete tumor regression and minimal toxicity in patients with vaginal or *para*-aortic recurrences treated with PBT (Uno et al., 2022, Yanazume et al., 2015; Mizuno et al., 2025). In the present study, we retrospectively evaluated the efficacy and toxicities of PBT for REC and investigated the predictors of local control (LC).

## 2 Methods

### 2.1 Patients

The medical records of patients with REC who received PBT at the Southern TOHOKU Proton Therapy Center from October 2008 to March 2021 were reviewed. Prior to PBT, computed tomography (CT) or fluorine-18-fluorodeoxyglucose positron emission tomography/CT (^18^F-FDG-PET/CT) was used to confirm the presence of no more than two lesions. Patients with REC who had previously received PBT were eligible for additional PBT if the recurrence was out of field and no more than two recurrent lesions were present. Exclusion criteria were as follows: (1) PBT administered for three or more recurrent lesions in a single course, (2) adjuvant PBT following local resection, (3) PBT for recurrence within a previously irradiated field after photon beam therapy or PBT, and (4) absence of follow-up imaging data for response evaluation.

### 2.2 Proton beam therapy

PBT was administered as part of standard clinical practice and not within the context of a prospective clinical trial. All patients were referred from outside institutions for consideration of PBT. Treatment indications were determined through discussion by a multidisciplinary cancer board, taking into account prior treatments, tumor location, proximity to organs at risk, and anticipated dosimetric advantages of PBT over photon-based RT.

CT with a 2.0-mm slice thickness was performed for PBT planning. Treatment planning was conducted using XiO-M (Hitachi, Kashiwa, Japan) with a Hitachi Proton Type Particle Therapy System (Hitachi). Gross tumor volume (GTV) was defined as the recurrent tumor. A median margin of 5 mm (range: 2–7 mm) was added around the GTV to generate the clinical target volume (CTV). An additional median margin of 5 mm (range: 3–10 mm) was applied to the CTV to form the planning target volume. PBT was performed at least 4–5 times per week using the passive scattering method. The typical dose-fractionation schedules at our institution were as follows: (1) 30–35 fractions of 1.8–2.0 Gy (relative biological effectiveness [RBE]) per fraction, or (2) 8–10 fractions of 6–8 Gy (RBE) per fraction, with a total dose of at least 60 Gy (RBE) in both regimens. The maximum allowable dose was 50 Gy (RBE) for the small intestine and 60 Gy (RBE) for the large intestine. Dose limits for the bladder, urethra, pelvic bones, and skin were set not to exceed the prescription dose. The final dose and fractionation plan were adjusted by the radiation oncologist in charge, based on factors such as tumor location, proximity to organs at risk, and the patient’s overall condition. An RBE value of 1.1 was applied, consistent with standard clinical practice in PBT. PBT was delivered without concurrent chemotherapy in all patients.

### 2.3 Outcomes

Treatment response was evaluated using post-PBT imaging studies, including CT or 18F-FDG-PET/CT. Response assessment on CT was performed Response Evaluation Criteria in Solid Tumors (RECIST) version 1.1 (Eisenhauer et al., 2009), whereas response on ^18^F-FDG-PET/CT was assessed according to the criteria established by the European Organisation for Research and Treatment of Cancer (Young et al., 1999).

LC was defined as the time from completion of PBT to the date of in-field progression. Progression free survival (PFS) was defined as the time from completion of PBT to the date of disease progression or death. Overall survival (OS) was defined as the time from completion of PBT to the date of death or last follow-up. The follow-up period was calculated from the date of PBT completion. PBT-related toxicities were evaluated using the Common Terminology Criteria for Adverse Events version 5.0. Acute and late toxicities were defined as adverse events occuring within 90 days and beyond 90 days after completion of PBT, respectively.

### 2.4 Statistical analysis

LC, PFS, and OS were analyzed using the Kaplan-Meier method. Potential prognostic factors influencing LC, such as histology, tumor size, irradiated site, pre-PBT maximal standardized uptake values (SUVmax) on ^18^F-FDG-PET/CT, irradiation dose, and treatment response, were evaluated. Cutoff values were determined using the receiver operating characteristic curve analysis, and the optimal cutoff value was defined as the point at which the sum of sensitivity and specificity was maximized. Univariate analyses were performed using the log-rank test. Statistical significance was set at *p* < 0.05. Statistical analyses were conducted using SPSS Statistics version 28.0 (IBM, Armonk, New York, USA).

## 3 Results

### 3.1 Patients

Ten patients with a total of 18 irradiated sites were treated with PBT between October 2008 and March 2021. Among 18 sites treated with PBT, four sites were excluded according to predefined exclusion criteria: (1) no patient was treated with PBT for three or more recurrent lesions in a single course; (2) one site (one patient) was treated with PBT after local resection; (3) two sites (one patient) were treated for in-field recurrence; and (4) 1 site (1 patient) lacked follow-up imaging for response evaluation. After these exclusions, nine patients with a total of 14 sites were included. Patient characteristics are summarized in Table 1**.** The median age at the time of initial PBT was 66 years (range, 42–79). According to the International Federation of Gynecology and Obstetrics 2008, six patients had stage I disease and three had stage III disease. The histological types were as follows: two cases each of endometrioid carcinoma, grade 1, endometrioid carcinoma, grade 3, clear cell carcinoma, and carcinosarcoma, and one case of adenocarcinoma. In this patient, pre-treatment biopsy confirmed adenocarcinoma of endometrial origin; however, detailed histopathological subtyping was unavailable. The median numbers of surgery and chemotherapy regimens were one (range, 1–3) and one (range, 1–5), respectively. Seven patients had received RT prior to PBT initiation. The numbers of sites treated with PBT per patient were as follows: five patients had one site, three patients had two sites, and one patient had three sites. The median intervals from the start of initial treatment to first recurrence and to initial PBT were 23.7 months (range, 8.7–164) and 44.3 months (range 17.4–165), respectively. The median follow-up period was 36.4 months (3.6–106.8).

**Table 1:** Clinical and treatment characteristics at initial proton beam therapy FIGO, International Federation of Gynecology and Obstetrics; NOS, not otherwise specified; PBT, proton beam therapy. One patient was classified as adenocarcinoma (NOS) because histopathological subtyping was unavailable.

		N = 9
Age (years)	Median (Range)	66 (42–79)
Stage (FIGO 2008)		
	I	6
	III	3
Histological type		
	Endometrioid carcinoma, grade 1	2
	Endometrioid carcinoma, grade 3	2
	Clear cell carcinoma	2
	Carcinosarcoma	2
	Adenocarcinoma, NOS	1
Number of pre-PBT surgery		
	1	6
	2	2
	3	1
Number of pre-PBT chemotherapy regimens		
	0	3
	1	3
	3	1
	5	1
Previous radiotherapy		
	Yes	7
	No	2
Time to first recurrence	Median (Range)	23.7 (8.7–164)
Time to first PBT	Median (Range)	44.3 (17.4–165)

The characteristics of the sites treated with PBT are shown in Table 2. Among the 14 sites, seven were lymph nodes; five were *para*-aortic nodes and two were pelvic nodes. Of the remaining seven sites, three were the vaginal stumps, three were the lungs, and one was the liver. Histological examination of the primary sites revealed endometrioid tumors at six sites and non-endometrioid tumors at seven sites. The median tumor size and SUVmax before PBT were 19.5 mm (range, 7–40) and 7.4 (range, 1.4–20.9), respectively. The median total dose was 66 Gy (RBE) (range, 60–70.4).

**Table 2:** Characteristics of sites treated with proton beam therapy SUVmax, maximum standardized uptake value; ^18^F-FDG-PET/CT, fluorine-18-fluorodeoxyglucose-positron emission tomography/computed tomography; PBT, proton beam therapy; RBE, relative biological effectiveness.

		N = 14
Histology		
	Endometrioid carcinoma, grade 1	3
	Endometrioid carcinoma, grade 3	3
	Clear cell carcinoma	5
	Carcinosarcoma	2
	Adenocarcinoma	1
Irradiated site		
	Lymph nodes	7
	Para-aortic	5
	Pelvic	2
	Vaginal vault	3
	Lung	3
	Liver	1
Tumor size (mm)	Median (range)	19.5 (7–40)
SUVmax of ^18^F-FDG-PET/CT	Median (range)	7.4 (1.4–20.9)
Interval from primary surgery to initiation of PBT (months)	Median (range)	44.3 (17.4–165)
Total dose (Gy, RBE)	Median (range)	66 (60–70.4)

### 3.2 Treatment and survival outcomes

**Fig. 1:**
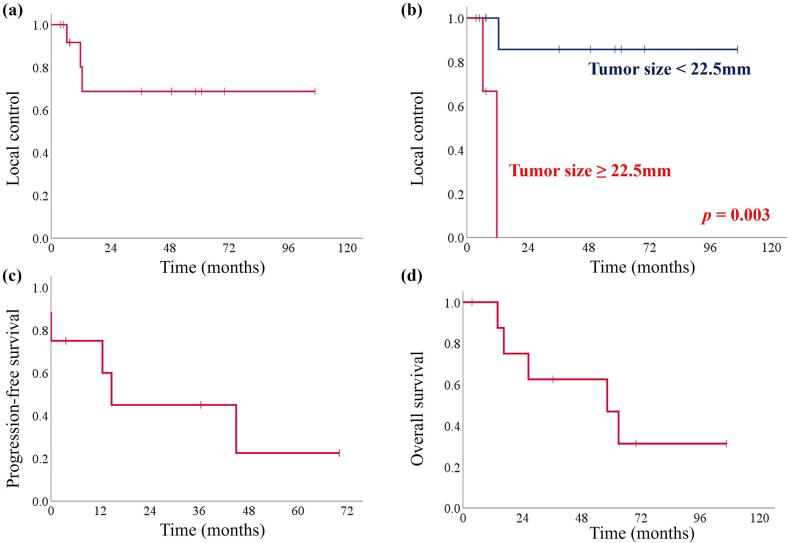
Kaplan-Meier survival curves. (a) Local control (LC), (b) LC stratified by tumor size (<22.5 mm vs. ≥22.5 mm), (c) Progression-free survival, (d) Overall survival.

Treatment response was assessed using post-treatment imaging, which was typically performed within 1–3 months (median, 2 months) after completion of PBT as part of routine clinical follow-up at our institution. Ten sites were assessed by CT and four sites by ^18^F-FDG-PET/CT. Among the ten sites evaluated by CT, eight showed complete response and two showed partial response. Of the four sites evaluated by ^18^F-FDG-PET/CT, three showed complete metabolic responses and one showed partial metabolic response. No patients received concurrent systemic therapy during PBT, and any subsequent systemic treatment was initiated only after disease progression. The 1-year and 2-year LC rates were 80.2 % and 68.8 %, respectively (Fig. 1a). In the univariate analysis, tumor size (cutoff value, 22.5 mm) was identified as a significant factor (*p* = 0.003). The 2-year LC rate was 85.7 % for lesions < 22.5 mm and 0 % for those ≥ 22.5 mm (Fig. 1b). In contrast, histology, irradiated sites, pre-PBT SUVmax on ^18^F-FDG-PET/CT, total radiation dose and treatment response were not significantly associated with LC (Table 3). The median PFS and OS were 14.7 months (range, 0–70.1) and 36.4 months (range, 3.6–106.8), respectively (Fig. 1, Fig. 1).

**Table 3:** Summary of univariate analysis results LN, lymph node; SUVmax, maximum standardized uptake value; ^18^F-FDG-PET/CT, fluorine-18-fluorodeoxyglucose-positron emission tomography/computed tomography; RBE, relative biological effectiveness; PBT, proton beam therapy; CR, complete response; CMR, complete metabolic response; CI, confidence interval; AUC, area under the curve.

Factor	Median	Range	Cut-off value	AUC	95 % CI	Group	n	p-value
Histlogy	−	−	−	−	−	Endometrioid	6	
(Endometrioid type or non-Endometrioid type)						non-Endometrioid	7	0.649
Tumor size	19.5	7–40	22.5	0.667	0.158–0.775	<22.5 mm	11	
(≥22.5 mm vs <22.5 mm)						≥22.5 mm	3	0.003
Irradiated site	−	−	−	−	−	non-LN	7	
(non-LN vs LN)						LN	7	0.174
SUVmax of 18F-FDG-PET/CT	7.4	3.9–25.1	4.1	0.574	0.214–0.934	<4.1	2	
(≥4.1 vs <4.1)						≥4.1	9	0.208
Total radiation dose	66	60–70.4	68	0.606	0.256–0.956	<68 Gy	8	
(≥68 Gy vs <68 Gy) (RBE)						≥68 Gy	6	0.551
Treatment response after PBT	−	−	−	−	−	CR + CMR	11	
(CR + CMR vs non-CR + non-CMR)						non-CR + non-CMR	3	0.083

### 3.3 Toxicity

Among the 14 treated sites, six developed grade 1 acute dermatitis. One patient developed grade 3 urinary tract infection associated with ureteral stenting. PBT was temporarily interrupted for several days, but the patient recovered after antibiotic therapy and subsequently completed the treatment. No GI or late adverse events attributable to PBT were observed.

## 4 Discussion

**Fig. 2:**
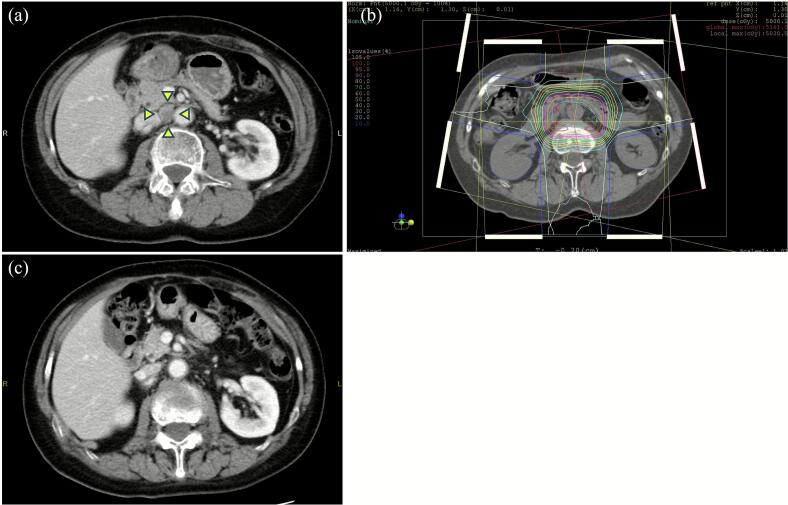
A representative case demonstrating the efficacy of proton beam therapy (PBT). This patient presented with a paraaortic lymph node metastasis that was treated with 66 Gy (RBE) using PBT. Fifty-eight months after treatment, this lesion remains in complete remission. (a) CT image showing the metastatic paraaortic lymph node before PBT (arrowheads). (b) Dose distribution of initial PBT treatment plan. (c) CT image obtained 2 months after completion of PBT. RBE, relative biological effectiveness; CT, computed tomography; PBT, proton beam therapy.

The present study demonstrated that PBT was an effective and well-tolerated treatment option for REC. The 1- and 2-year LC rates were 80.2 % and 68.8 %, respectively, with the median PFS and OS of 14.7 and 58.4 months. Tumor size < 22.5 mm was associated with favorable outcomes, achieving a 2-year LC rate of 85.7 %. Treatment-related toxicities were minimal, with only one patient developing grade 3 acute urinary tract infection. A representative case treated with PBT is presented in Fig. 2.

Our results demonstrated promising LC and survival outcomes, despite the clinical heterogeneity and a high proportion of patients with prior RT. In the GOG-238 trial, patients with isolated vaginal cuff recurrence and no prior RT achieved a 3-year LC rate of 65 % and a median PFS of 20.7 months following external-beam RT with or without concurrent cisplatin. (Klopp et al., 2024). Despite including heavily pretreated patients and extra-pelvic recurrences, our study achieved a 2-year LC rate of 68.8 %, suggesting that PBT may offer comparable efficacy in a more challenging population. Similarly, Lindemann et al. reported a 2-year LC rate of 77 % and a median OS of 34 months in a clinically heterogeneous cohort of patients with central pelvic or vaginal relapse treated with salvage photon-based RT; notably, all patients were RT-naïve at the time of salvage treatment. These findings are consistent with our results (Lindemann et al., 2021). Taken together, these comparisons further underscore the robustness of our study, particularly given that 78 % of our patients had undergone prior RT and several presented with extra-pelvic recurrences.

Furthermore, McAlarnen et al. reported favorable outcomes with multimodal salvage strategies, including surgery, chemotherapy, and photon-based RT, achieving 2-year PFS and OS rates of 51 % and 69 %, respectively (McAlarnen et al., 2019). While our study demonstrated the efficacy of PBT alone, these findings suggest that integrating PBT with systemic therapies may further enhance outcomes, particularly in patients with disseminated diseases.

In the present study, tumor size emerged as the only significant predictor of LC following PBT, with lesions < 22.5 mm demonstrating markedly higher 2-year LC rates than larger tumors (85.7 % vs. 0 %). This finding is consistent with the report by Wylie et al., who showed significantly poorer LC for tumors > 2 cm after salvage RT for REC (Wylie et al., 2000). Similar associations between smaller tumor size and improved LC have also been described in other malignancies treated with PBT (Yamaguchi et al., 2023, Shin et al., 2021, Chadha et al., 2019, Yang et al., 2024). A plausible explanation is that larger tumors are more likely to harbor hypoxic regions and exhibit reduced dose uniformity and increased intratumoral heterogeneity, all of which result in decreased radiosensitivity (Alfonso and Berk, 2019; Beckers et al., 2024). While these mechanisms have primarily been investigated in photon-based therapy, they are probably applicable to PBT as well. From clinical standpoint, tumor size is an objective and readily available parameter and may therefore serve as a practical criterion for selecting patients most likely to benefit from PBT.

PBT demonstrated a favorable safety profile in the present study. Among 14 treatment courses, six patients developed only grade 1 acute dermatitis, and no GI toxicities were observed. One patient experienced a grade 3 urinary tract infection, which occurred in the context of ureteral stenting and was considered unlikely to be directly related to PBT. Despite prior RT in most patients, no late adverse events were reported. These outcomes compare favorably with those of photon-based RT, including IMRT, where grade ≥ 3 GI toxicities have been reported in up to 8–10 % of cases (Klopp et al., 2024; Ho et al., 2015). In addition, the prospective phase II APROVE trial demonstrated that postoperative pelvic PBT for cervical and endometrial cancer patients was feasible and well tolerated, with no grade ≥ 3 GI and genitourinary toxicities (Arians et al., 2023, Meixner et al., 2023). Furthermore, patient-reported outcomes at long-term follow-up indicated recovery of global health status and overall quality of life. Although conducted in a different clinical setting, these findings are consistent with the favorable toxicity profile observed in our salvage cohort. The unique physical properties of PBT, particularly the Bragg peak, allow for sparing of adjacent organs at risk and likely account for the reduced toxicity observed. Collectively, these findings support the role of PBT as a safe and effective salvage modality for REC.

The present study has several limitations. First, the sample size was small, limiting the statistical power and precluding robust subgroup analyses. Second, the retrospective, single-institutional design may have introduced selection and information bias. Third, heterogeneity in recurrence sites and treatment parameters may have affected the interpretation of outcomes. Despite these limitations, this study provides valuable preliminary evidence supporting the feasibility and safety of PBT for REC.

## 5 Conclusions

This study demonstrated that PBT is a safe and potentially effective salvage treatment for REC, even in heavily pretreated patients. LC rates were favorable, and treatment was well tolerated with minimal toxicity. Tumor size emerged as a significant predictor of LC, with smaller tumors achieving substantially better outcomes. These findings suggest that PBT may be particularly suitable for patients with limited-volume recurrence, especially when lesions are located near radiosensitive structures.

## CRediT authorship contribution statement

**Yuta Endo:** Writing – review & editing, Writing – original draft, Visualization, Methodology, Investigation, Formal analysis, Data curation. **Yoshiaki Takagawa:** Writing – review & editing, Investigation, Formal analysis. **Yuki Yoshimoto:** Investigation, Formal analysis. **Koki Ando:** Investigation, Formal analysis. **Rei Nishikawa:** Investigation, Formal analysis. **Masanori Machida:** Investigation, Formal analysis. **Yuntao Dai:** Investigation, Formal analysis. **Ichiro Seto:** Investigation, Formal analysis. **Motohisa Suzuki:** Investigation, Formal analysis. **Takahiro Kato:** Investigation, Formal analysis. **Shigenori Furukawa:** Investigation, Formal analysis. **Shu Soeda:** Investigation, Formal analysis. **Keiya Fujimori:** Investigation, Formal analysis. **Masao Murakami:** Supervision.

## Ethics approval and consent to participate

This study was approved by the Ethics Committee of Southern TOHOKU Research Institute for Neuroscience (No. 565) and was announced on the website of Southern TOHOKU Proton Therapy Center and Southern TOHOKU General Hospital. Hospital. The research was conducted in accordance with the 1964 Helsinki Declaration. The study was conducted using the opt-out method. As this was a retrospective study, some patients had already deceased or discontinued follow-up.

## Funding

Not applicable.

## Declaration of competing interest

The authors declare that they have no known competing financial interests or personal relationships that could have appeared to influence the work reported in this paper.

## Data Availability

The datasets used and/or analyzed in the current study are available from the corresponding author upon reasonable request.
